# The Pocket Anatomist; Being a Complete Description of the Anatomy of the Human Body

**Published:** 1861-01

**Authors:** 


ART. IX.—*The Pocket Anatomist, being a Complete Description of the Anatomy of the Human Body. For the Use of Students.* By M. W. HILLES, formerly Lecturer on Anatomy and Physiology at the Westminster Hospital School of Medicine. 12mo., pp. 263. Philadelphia: Lindsay & Blakiston, 1860.

MR. HILLES, who has for some time been favorably known to us by his excellent little work on Regional Anatomy, issued in London, in 1857, now offers for the use of students the most complete book of the kind we have ever seen. It is not intended to take the place of the larger and more finished text-books, but the author's aim has been to furnish a brief description of the different tissues and organs of the human body. As stated in the preface, the work is based upon a treatise known to the profession as *Savage's Anatomist*, and from its enlarged and altered form, it should be considered rather as a new work than as a new edition of a former treatise. It is vastly superior, in every respect, to the Anatomical Remembrancer, so much used in this country, and we can, therefore, safely recommend it to the attention of students.

